# Supplementary material for: Nanoscale kinetic segregation of TCR and CD45 in engaged microvilli facilitates early T cell activation
Source: Nat Commun. 2018 Feb 21;9:732. doi: 10.1038/s41467-018-03127-w (PMC5821895; doi:10.1038/s41467-018-03127-w)
Supplement: Supplementary file 1 — Supplementary Information [file 41467_2018_3127_MOESM1_ESM.pdf]

Supplementary Figure 1

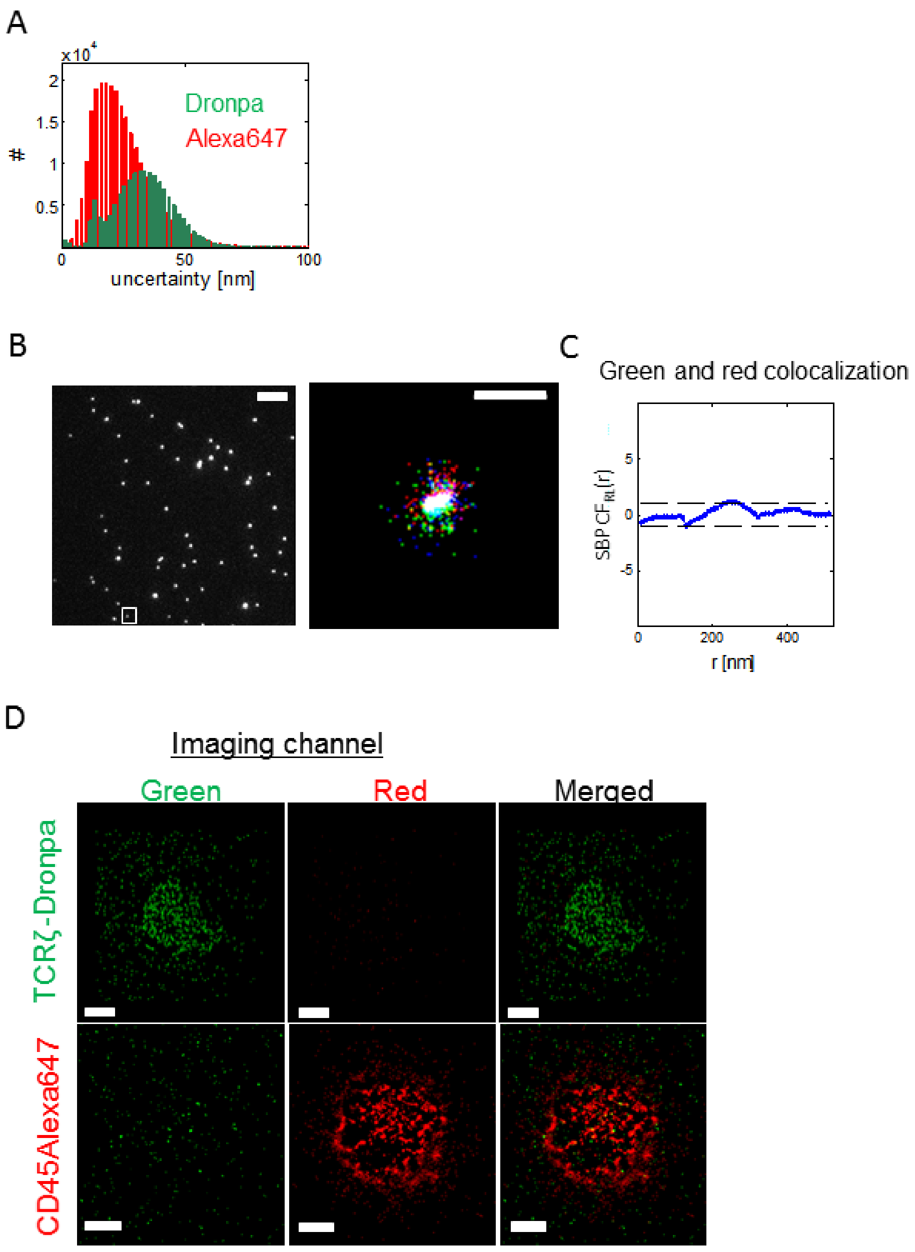

Supplementary Figure 1. SMLM imaging characteristics

(A) Histograms of the localization uncertainty obtained in the SMLM PALM combined dSTORM imaging of Dronpa (green histogram) and Alexa647 (red histogram). (B) Widefield (left; scale bar 5 µm) and zoom (right; scale bar 500 nm) images of 100 nm multicolour TetraSpeck beads (Life Technologies) taken in SMLM mode in 3 channels.

(C) Colocalization analysis using SBPCF statistics (BPCF, normalized by the RL model; see details in Materials and methods). (D) Multicolour SMLM (PALM combined dSTORM) image of Jurkat E6.1 cells expressing TCRζ-Dronpa (Upper panel) or immune-stained for CD45 with Alexa647 (lower panel). The cells were fixed after 4 min after dropping onto αCD3-coated coverslips. Scale bar - 2 μm.

Supplementary Figure 2

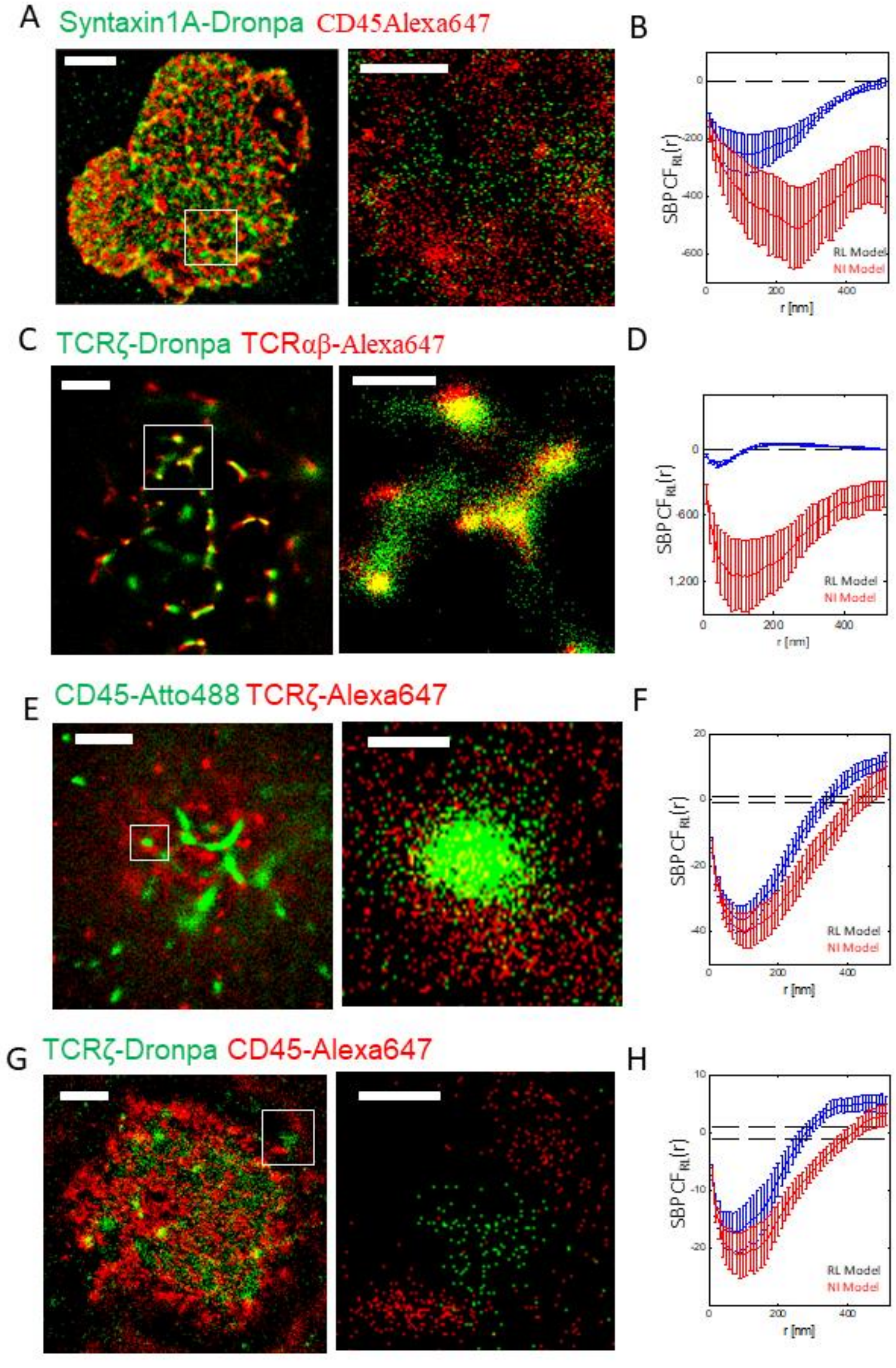

Supplementary Figure 2. Controls for SMLM imaging of bimolecular protein organization

(A) Two-colour SMLM, PALM combined dSTORM imaging of Jurkat E6.1 cells expressing Syntaxin1A-Dronpa (green) and CD45 immunostained with an αCD45-primary antibody conjugated to Alexa647 (red). (B) SBPCF statistics of Syntaxin1A and CD45 for multiple cells (in blue), compared to 95% confidence intervals due to models of no-interaction (NI; red) and random labelling (RL; black). N=14 cells. (C) Two-colour SMLM, PALM combined dSTORM imaging of Jurkat E6.1 cells expressing TCRζ-Dronpa (green) and TCRαβ immunostained with Alexa647 (red). (D) SBPCF statistics of TCRζ and TCRαβ for multiple cells (N=12). (E) Two-colour dSTORM imaging of Jurkat E6.1 cells immunostained for CD45 by Atto488 (green) and for TCRζ by Alexa647 (red). (F) SBPCF statistics of TCRζ and TCRαβ for distinct contact areas in multiple cells (N=14 cells; n=47 areas). (G) Two-colour SMLM, PALM combined dSTORM imaging of Jurkat E6.1 cells expressing TCRζ-Dronpa (green) and CD45 immunostained with Alexa647 (red). (H) SBPCF statistics of TCRζ and CD45 for distinct contact areas in multiple cells (N=15 cells; n=48 areas). In all panels (A-H) the cells were dropped on αCD3-coated coverslips and fixed after 4 min. Scale bars - 2 μm (left) and 500nm (right).

Supplementary Figure 3

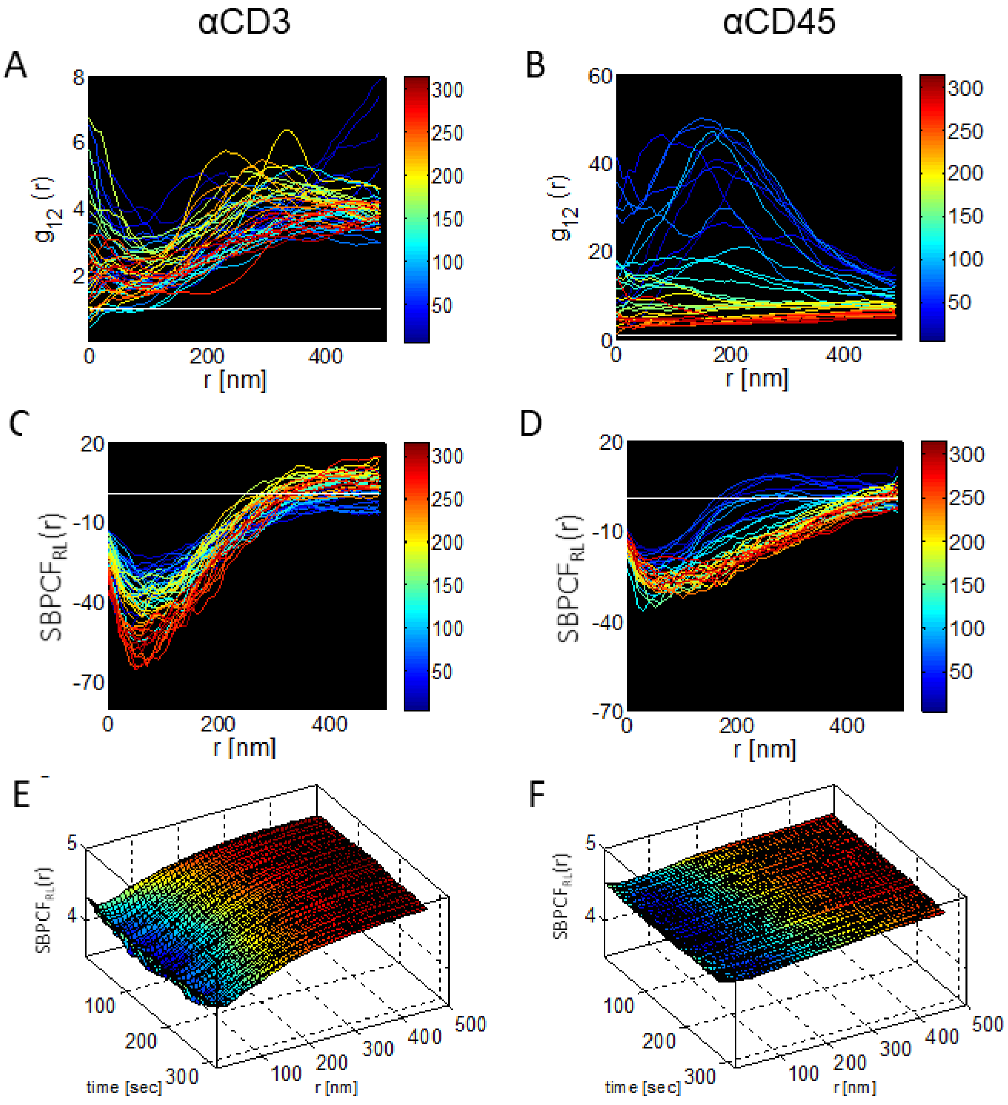

Supplementary Figure 3. The dynamics of the physical separation of TCR and CD45

(A,B) PCF curves of TCR-CD45 molecules of Jurkat E6.1 cells expressing TCRζ-Dronpa and immunostained for CD45 following a cell spreading on either an (A) αCD3 or (B) αCD45-coated coverslips. The colours depict the time point for which the PCF curves were calculated (dark blue represents the start of the cell spreading process and dark red represents 300 sec later). (C,D) The PCF curves from panel A,B normalized by the RL model. Colour coding is identical to the one used in panels A and B. (E,F)

The PCF curves normalized by the RL model and plotted as a 2D manifold for cells on (E) αCD3 or (F) αCD45-coated coverslips. The colours depict the height of the normalized PCF curves (dark blue corresponds to low levels of the PCF and dark red correspond to high levels of the PCF). This figure relates to Fig. 1 in the main text.

Supplementary Figure 4

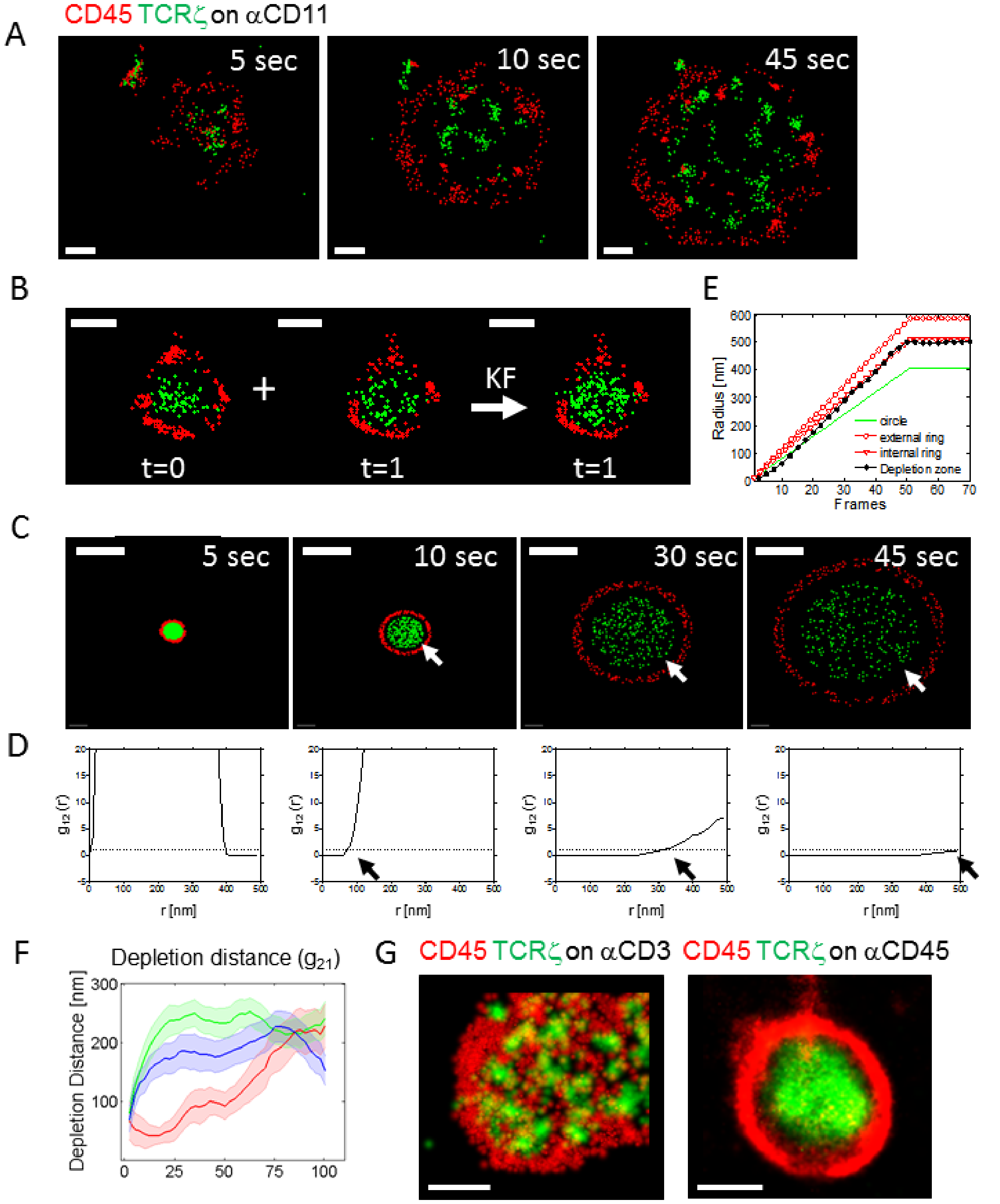

Supplementary Figure 4. Analyses of the kinetic segregation and the depletion zone in early forming contacts

(A) Time sequence of the synapse formed at a distinct area (zoom view) within the PM of Jurkat E6.1 cells expressing TCRζ-Dronpa (green) and immunostained for CD45 with Alexa647 (red) on an αCD11a-coated coverslip. (B) Live cell PALM combined

dSTORM time position of TCR (green) and CD45 (red) before and after applying Kalman filtering with variance estimate of acquisition noise of 0.05 and bias of the prediction of 0.5. (C) Model simulation of a ring recedes from an inner circle in time. Both the circle and the ring are growing linearly but with different rates. Arrows points to the positions were a depleted zone start to form. Scale bar - 1 µm. (D) The corresponding PCF of the circle' molecules with the ring' molecules. Arrows points to the positions were the PCF crosses the value of one, that is, the defined depletion zone value. (E) The growth rates of the circle (green line), the outer (red circles) and inner (red squares) ring's walls and of the depletion zone value (black filled circles). (F) The depletion distance calculated by the PCF between CD45 molecules and TCR molecules (g_{12}) under various conditions. (G) Accumulated (sum) images of the areas analysed in Fig. 2A. This figure relates to Fig. 2 in the main text.

Supplementary Figure 5

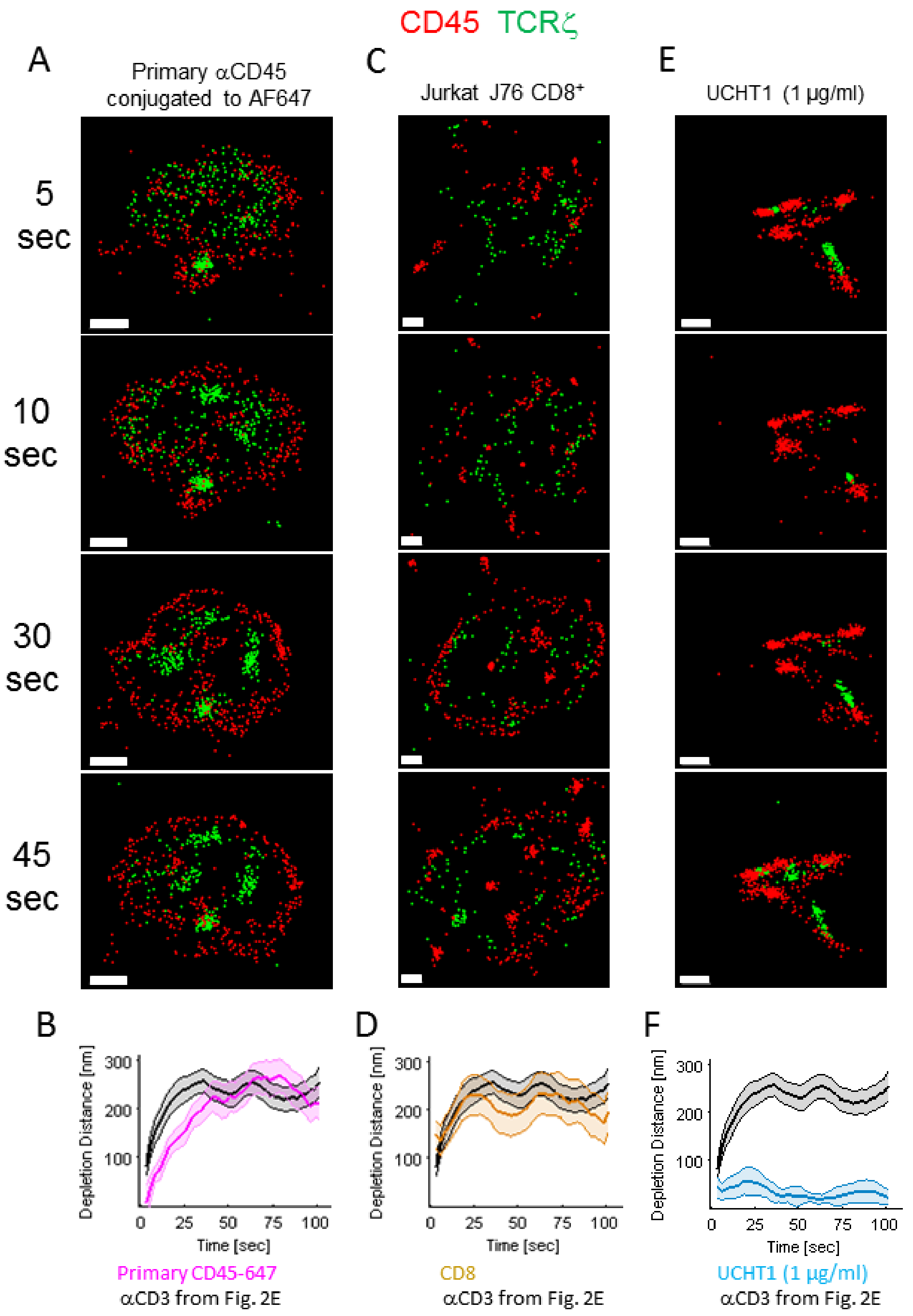

Supplementary Figure 5. The evolution of a depletion zone and its dynamics under additional conditions

(A) Time sequence of the synapse formed at distinct areas between Jurkat E6.1 cells expressing TCRζ-Dronpa (green) and immunostained with a primary αCD45 conjugated to Alexa647 (red) on coverslips coated with αCD3ε (UCHT1; 10 μg/ml).

Scale bar – 0.5 μm. (B) The depletion distance between TCR and CD45 molecules for multiple cells, as in panel A (N=19). (C) Time sequence of the synapse formed at distinct areas between Jurkat J76 ($CD8^+$) cells expressing TCRζ-Dronpa (green) and immunostained with a primary αCD45 and a secondary, conjugated to Alexa647 (red) on coverslips coated with αCD3ε (UCHT1; 10 μg/ml). Scale bar – 0.5 μm. (D) The depletion distance between TCR and CD45 molecules for multiple cells, as in panel C (N=11). (E) Time sequence of the synapse formed for Jurkat E6.1 cells labelled and imaged as in panel C on coverslips coated with αCD3ε (UCHT1; 1 μg/ml). Scale bar – 0.5 μm. (F) The depletion distance between TCR and CD45 molecules for multiple cells, as in panel E (N=12). (B,D,F) Results (in coloured lines) are compared between data from the relevant panels in this figure with data from Fig. 2E (αCD3; in black). This figure relates to Fig. 2 in the main text.

Supplementary Figure 6

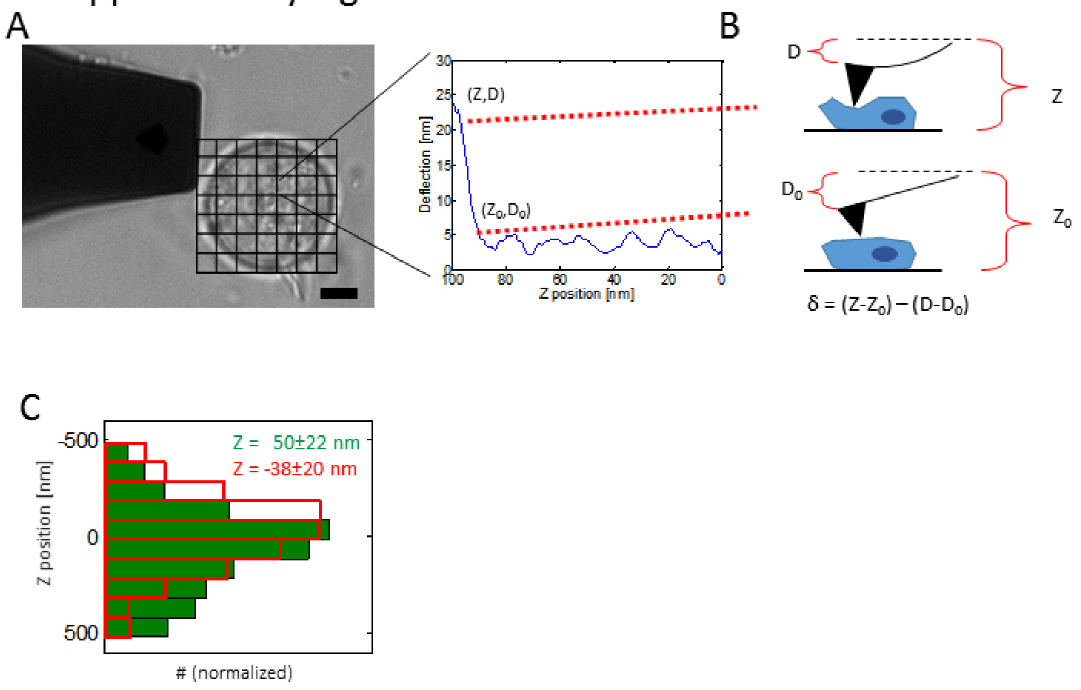

Supplementary Figure 6. Quantitative Imaging AFM and 3D SMLM statistics

(A) Quantitative Imaging (QI^{TM}) experiment is done by conducting a force curve on every pixel separately over the entire cell. A representative force curve is present. Z and D are abbreviations for the AFM cantilever position in the z axis and its deflection. Z_0 and D_0 are the cantilever position and deflection at its contact point with the cell. Scale bar 2 µm. (B) Indentation experiment illustration. During a force curve the tip indent into the cell and deforms it. The definition of the indentation distance δ is shown. (C) Histogram of z heights of detected TCRζ and CD45 in footprints of multiple cells (N=5), as in Fig. 3H. TCR represented by full green bars and CD45 by empty red bars. This figure relates to Fig. 3 in the main text.

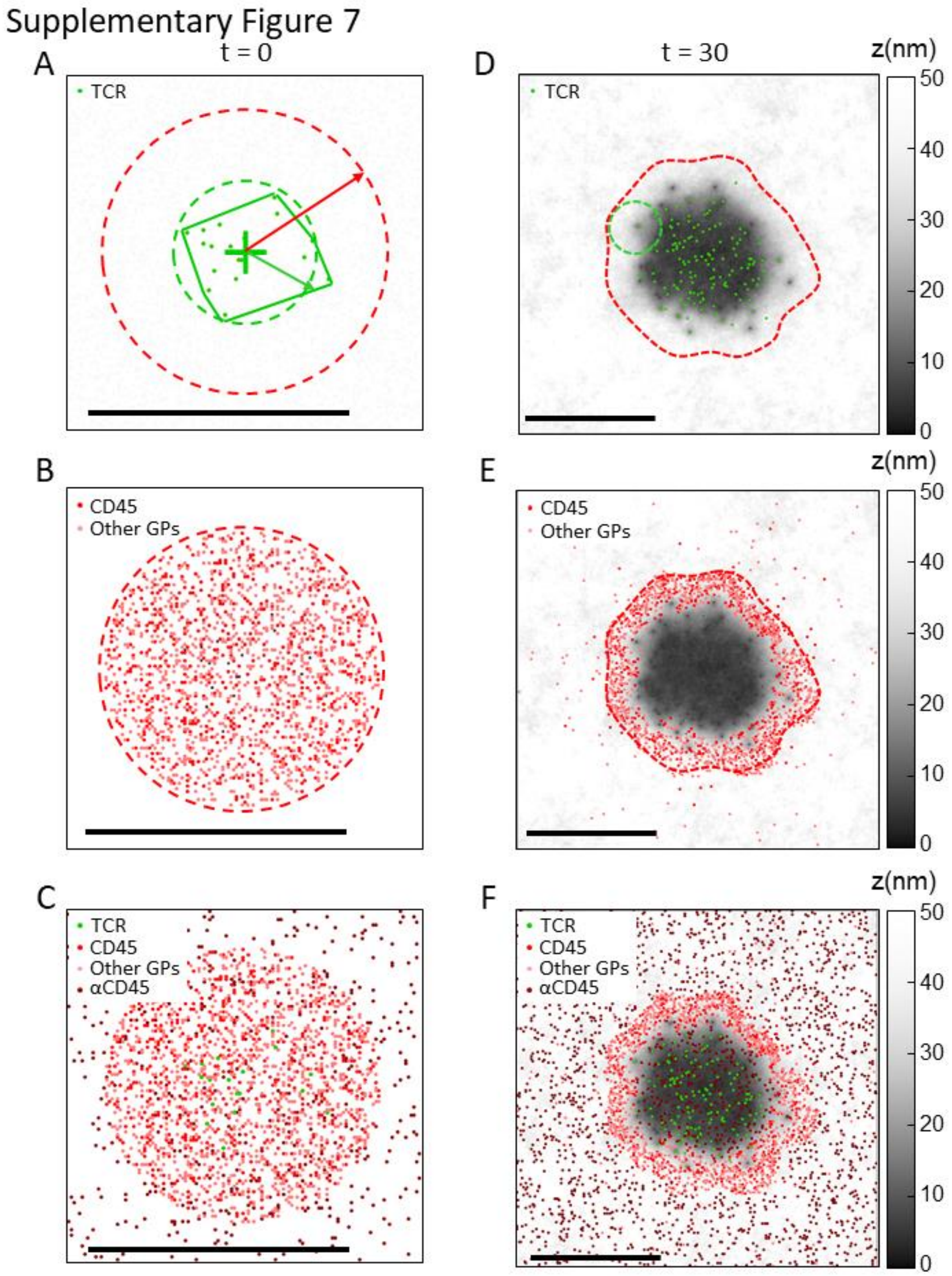

Supplementary Figure 7. Initial conditions of the simulations

(A) Initial simulation conditions. The TCRs locations (green points) at t = 0 sec are set by the first frame of the experimental movie, taken at t = 2.5 sec. To scatter the glycoproteins at t = 0 we use the TCRs locations at t = 5 sec (i.e. the second frame of the experimental movie). The TCR points are divided into clusters if they are separated

by more than 300 nm. For each cluster the green convex polygon contains all of its TCR points. The green cross is the mean of the X and Y coordinates of the TCRs. The radius of the green dashed circle (TCR circle) is the square root of the polygon area. The radius of the red dashed circle (the initial GPs circle) is set to 2x(radius of green circle). Scale bar - 1 μm. (B) The number of CD45 molecules scattered at t = 0 sec equal the average number of experiment CD45 taken from t = 10 sec to t = 60 sec and remains the same throughout the simulation. The CD45 (red points), and other GPs (pink points) molecules are scattered uniformly inside the red circle. Every 10x10 nm pixel can accommodate only one molecule. (C) Merged field of TCRs and glycoproteins, as set in panels A and B with the αCD45 (brown points) that are uniformly scattered at the given ligand density.

(D) Maximal apparent separation of TCRs and glycoproteins (GP). Around every TCR point we draw a circle with a radius of maximal apparent separation (green dashed circle), the sum of all the individual separations creates the GP maximal apparent separation (red dashed contour). In the simulation GPs inside the contour cannot jump out of it. GPs outside the contour can jump in. GPs can get outside if at the initial conditions they were already outside the contour or if the contour shifts during the simulation. Scale bar – 1 μm. (E) The mobility of the CD45 is restricted because of the binding between CD45 and αCD45, while the mobility of the other GP molecules is not interrupted. Scale bar – 1 μm. (F) Merged field of TCRs and glycoproteins, as set in panels D and E and the αCD45 ligands.

Supplementary Figure 8

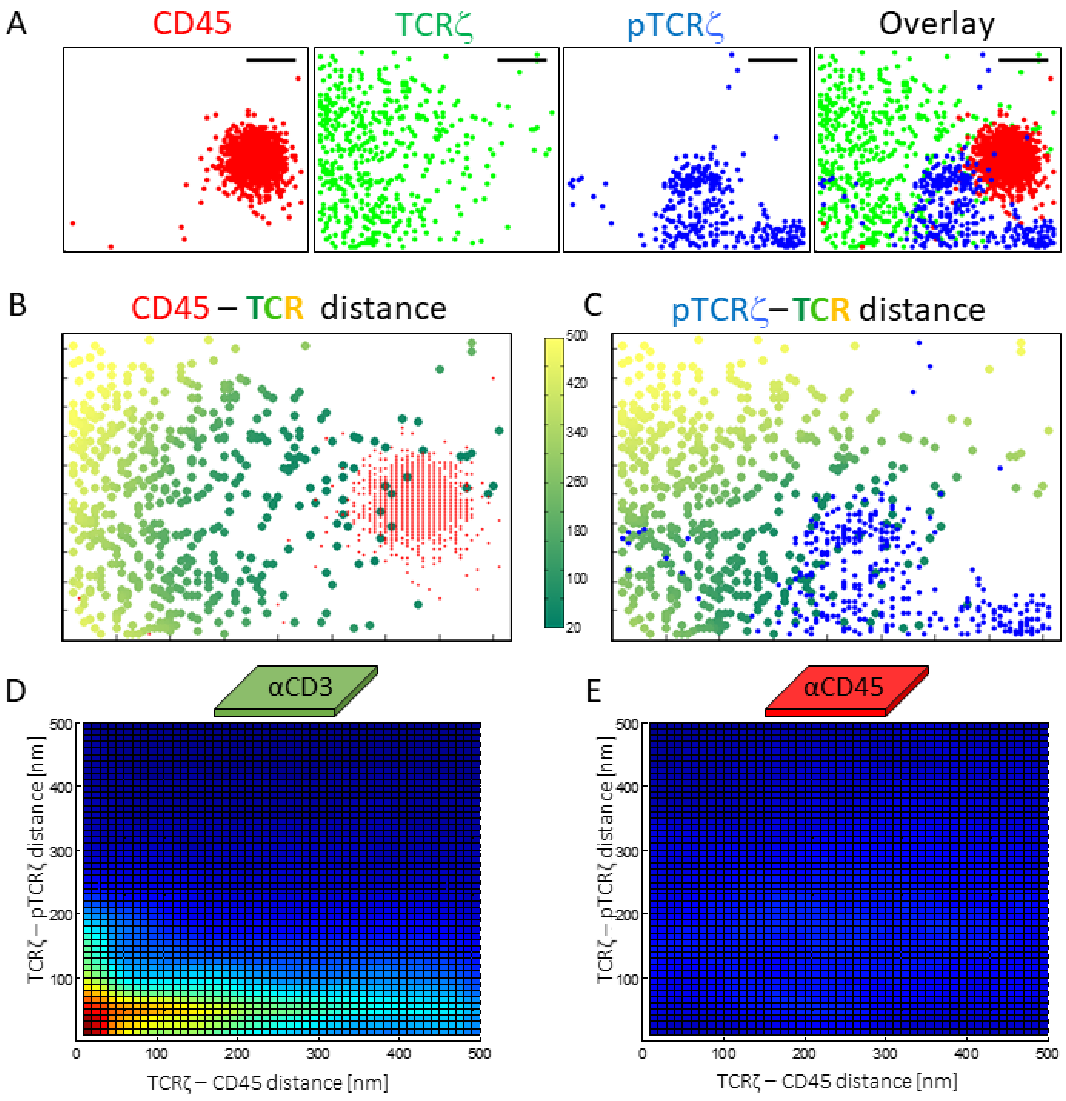

Supplementary Figure 8. Correlation maps between TCR-CD45 and TCR-pTCR

(A) Multicolour SMLM PALM combined dSTORM panel of Jurkat E6.1 expressing TCR$_\zeta$-Dronpa (green) and immunostained for CD45 (red) and pTCR$_\zeta$ (blue) with Alexa568 and Alexa647 correspondingly, fixed after 4 min of spreading on an αCD3 coverslip. (B) TCR molecules are coloured by the nearest neighbours distance score from CD45 molecules. Dark green correspond to small distance value and light green correspond to high distance value. (C) TCR molecules are coloured by the nearest neighbours distance score from pTCR$_\zeta$ molecules. Dark green correspond to small

distance value and light green correspond to high distance value. (D,E) 2D histograms of the two TCR's scoring maps, the distance from CD45 and from pTCRζ maps, of cells spread on either (D) αCD3- or (E) αCD45-coated coverslips. This figure relates to Fig. 6 in the main text.

Supplementary Figure 9

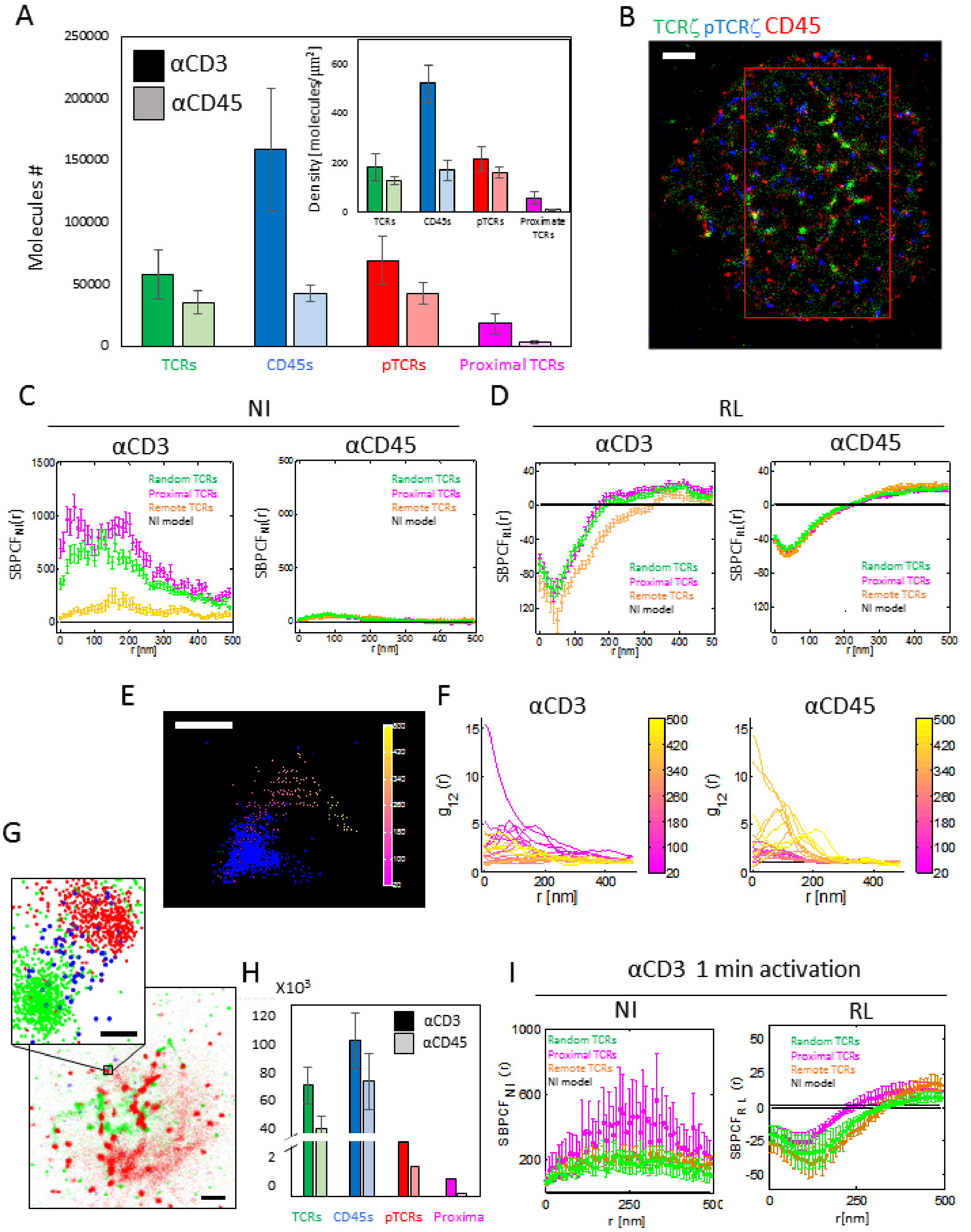

Supplementary Figure 9. Complementary analyses of TCR phosphorylation as a function of the depletion between CD45 and the TCR

(A) Molecular counts and density (inset) of TCRζ (green), CD45 (blue), pTCRζ (red) and proximal TCR (magenta) molecules of Jurkat E6.1 cells expressing $TCR_{ζ}$-Dronpa and immunostained for CD45 and pTCRζ with Alexa568 and Alexa647 respectively. The cells were fixed after 4 min from dropping on an αCD3 (n=12) or αCD45 (n=14) coverslips. (B) A typical region of interest selected for conditional second order statistics analysis. Scale bar 2 µm. (C,D) Conditional bivariate PCF analyses between TCR and CD45 molecules of Jurkat E6.1 cells fixed after 4 min from dropping on an αCD3 or αCD45 coverslips normalized by either (C) the NI model or (D) the RL model. (E) TCR molecules ('spring' colourmap) are coloured by their distance from pTCR (blue). Magenta-to-yellow colour gradient correspond to short-to-long distances respectively, scale bar 500 nm. (F) The PCF of the subpopulation of TCR with CD45 molecules of cells spread on an αCD3 (left) or αCD45 (right) coverslips. The colour of the PCF correspond to the TCR subpopulation distance from pTCR molecules as depicted in (E). (G) Multicolour SMLM, PALM combined dSTORM image of Jurkat E6.1 expressing TCRζ-Dronpa (green) and immunostained for CD45 (red) and pTCRζ (blue) with Alexa568 and Alexa647 respectively, and fixed after 4 min of spreading on an αCD3-coated coverslips. Scale bar 2 µm. Inset shows a zoom region. Scale bar 200 nm. (H) Molecular counts of TCRζ (green), CD45 (blue), pTCRζ (red) and proximal TCR (magenta) molecules for multiple cells, as in panel G. The cells were fixed after 1 min from dropping onto coverslips coated with either αCD3 (N=13) or αCD45 (N=12). (I) Conditional bivariate PCF analyses between TCR and CD45 molecules of cells, as in panel G, normalized by either the no-interaction (NI) model (left) or the random labelling (RL) model (right). This figure relates to Fig. 6 in the main text.

Supplementary Figure 10

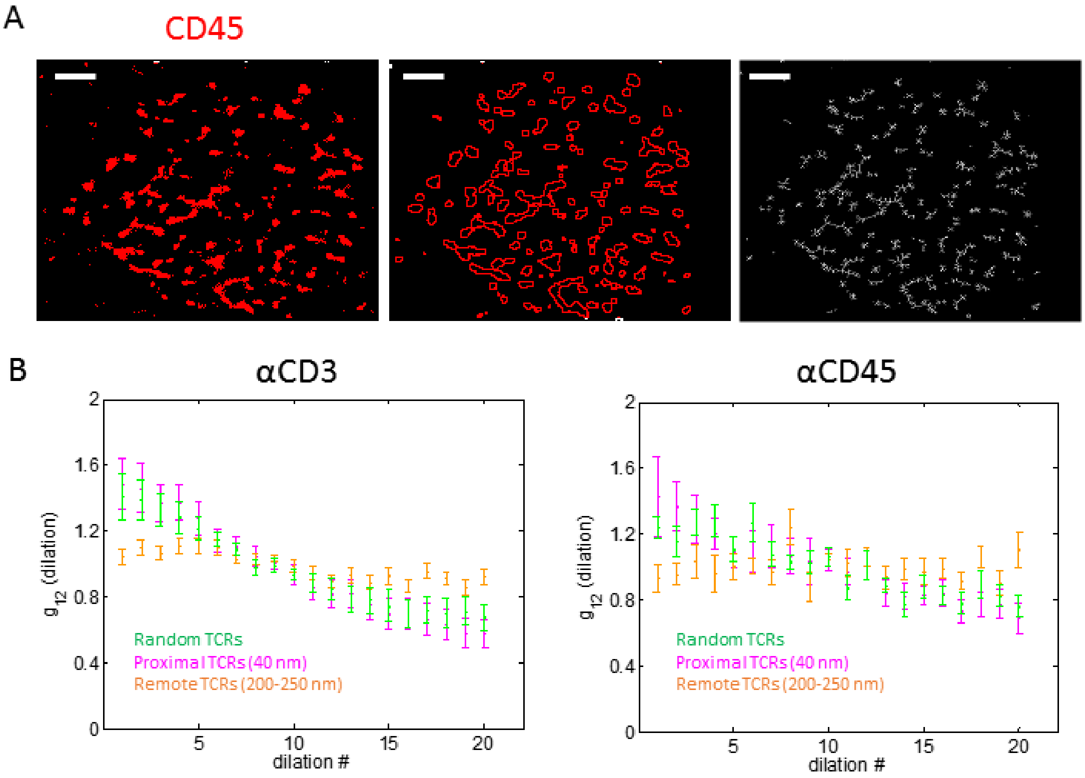

Supplementary Figure 10. Morphological analyses of TCR activation in single clusters

(A) (left). Scatter plot of CD45 molecules of a representative Jurkat E6.1 cell fixed after 4 min from dropping on an αCD3-coated coverslip. (middle). Contour map (contour level 2% of all contours) of CD45 density map. (right). Skeleton map of the contour generated by Matlab morphological operations tool box, BWMorph. Scale bar 2 µm. (B) A conditional bivariate PCF analysis adopting the dilation shapes as a basis for the spatial statistics between TCR and CD45 molecules of Jurkat E6.1 cells fixed after 4 min from dropping on αCD3-coated (left) or αCD45-coated (right) coverslips. This figure relates to Fig. 7 in the main text.

Supplementary Table 1 - Differences between experiments and simulations

**Property**	**Experiment**	**Simulation**	**Fig.**	**Ref.***
Array size [µm]	2.5 – 4.5	Taken from experiment	4D,E	
Boundary conditions	Non periodic	periodic		5,20
Frame time [sec]	2.5	0.01	4,5	
Number of TCRs	Changes with time	Taken from experiment	4D,E	
Number of CD45	Changes with time	Constant, mean of experiment (t = 10 – 60 sec)	4D,E	
Number of other GPs	-	~2x the no. of CD45		1,12
Locations of TCRs (t = 0)	Taken from t = 2.5 sec	Taken from experiment	SF7	
Locations of CD45 (t = 0)	-	Circle around TCR cluster	SF7	
Locations of other GPs (t = 0)	-	Circle around TCR cluster	SF7	
Locations of TCRs (t > 0)	Measured	Taken from experiment	4D,E, SF7	
Locations of CD45 (t > 0)	Measured	Simulated	4D,E, SF7	
Locations of other GPs (t > 0)	-	Simulated	SF7	
Membrane height, z [nm]	-	0 - 50	4, SF7	5
z(t = 0) [nm]	-	50	SF7	
z(TCRs) [nm]	-	13	4B,C	1,12
TCR-αCD3 interactions	-	Inferred from TCR locations in experiment	4D,E, SF7	
Repulsive k of GPs [K_BT nm^{-2}]	-	0.025	4	20
Repulsive potential of GPs [K_BT]	-	$0.5*k(50 – z)^2$	4	20
αCD3 density [# µm^{-2}]	-	NA (TCR locations are taken from experiment)	5B	5
αCD45 density [# µm^{-2}]	-	0,300,1000,3000,10000	5D	5
Membrane rigidity [K_BT]	7.75±3	3,10,25,100	5,SF6 A,B	20
Maximal apparent separation [nm]	-	100,150,200,250,300,500	4D,E, 5B,D	

Comment: ***** References from main text
