# Supplementary material for: Nanoscale kinetic segregation of TCR and CD45 in engaged microvilli facilitates early T cell activation
Source: Nat Commun. 2018 Feb 21;9:732. doi: 10.1038/s41467-018-03127-w (PMC5821895; doi:10.1038/s41467-018-03127-w)
Supplement: Supplementary file 3 — Description of Additional Supplementary Files [file 41467_2018_3127_MOESM3_ESM.pdf]

Description of Additional Supplementary Files

File Name: **Supplementary Movie 1**

Description: **Physical separation of TCR and CD45 in live cell spread on αCD3**

A representative movie of two-colour SMLM, PALM combined dSTORM of live Jurkat E6.1 cells expressing TCRζ-Dronpa (green) and CD45 immunostained with Alexa647 (red) spread on an αCD3-coated coverslip. Movie is part of the data presented in Fig. 1B left panel. The movie is played at 800 frames per second (accumulation of 10 sec). Scale bar – 2 μm.

File Name: **Supplementary Movie 2**

Description: **Physical separation of TCR and CD45 in live cell spread on αCD45**

A representative movie of two-colour SMLM, PALM combined dSTORM of live Jurkat E6.1 cells expressing TCR ζ-Dronpa (green) and CD45 immunostained with Alexa647 (red) spread on an αCD45-coated coverslip. Movie is part of the data presented in Fig. 1B right panel. The movie is played at 800 frames per second (accumulation of 10 sec). Scale bar – 2 μm.

File Name:**Supplementary Movie 3**

Description: **The evolution of a depletion zone at microvilli tips on αCD3**

A representative movie of a newly forming region (zoomed view), imaged using two-colour SMLM, PALM combined dSTORM of live Jurkat E6.1 cells expressing TCRζ-Dronpa (green) and CD45 immunostained with Alexa647 (red) spread on an αCD3-coated coverslip. Movie is part of the data presented in Fig. 2A left panel. The movie is played at 800 frames per second (accumulation of 10 sec). Scale bar – 0.5 μm.

File Name: **Supplementary Movie 4**

Description: **The evolution of a depletion zone at microvilli tips on αCD45**

A representative movie of a newly forming region (zoomed view), imaged using two-colour SMLM, PALM combined dSTORM of live Jurkat E6.1 cells expressing TCRζ-Dronpa (green) and CD45 immunostained with Alexa647 (red) spread on an αCD45-coated coverslip. Movie is part of the data presented in Fig. 2A right panel. The movie is played at 800 frames per second (accumulation of 10 sec). Scale bar – 0.5 μm.
